# Prognostic impact of lymph node parameters in distal cholangiocarcinoma after pancreaticoduodenectomy

**DOI:** 10.1186/s12957-020-02040-1

**Published:** 2020-10-08

**Authors:** Shaocheng Lyu, Lixin Li, Xin Zhao, Zhangyong Ren, Di Cao, Qiang He

**Affiliations:** Department of Hepatobiliary Surgery, Beijing Chaoyang Hospital, Capital Medical University, 8 Gongtinan Road, Chaoyang District, Beijing, 100020 China

**Keywords:** Cholangiocarcinoma, Pancreatoduodenectomy, Lymph node metastasis, Survival

## Abstract

**Background:**

Pancreaticoduodenectomy is the only definitively curative therapy for the long-term survival of distal cholangiocarcinoma patients. Lymph node metastasis is widely accepted as an important prognostic factor for distal cholangiocarcinoma. The latest American Joint Committee on Cancer (AJCC) TNM classification system for distal cholangiocarcinoma has divided the lymph node metastasis patients into N1 and N2 by lymph node metastasis number. However, some studies suggested that the lymph node metastasis ratio may be better than the lymph node metastasis number. Therefore, we develop a program to analyze the correlation between lymph node parameters (lymph node dissection number, lymph node metastasis number, and lymph node metastasis rate) and long-term prognosis.

**Methods:**

We retrospectively reviewed 123 distal cholangiocarcinoma patients after pancreatoduodenectomy from January 2011 to December 2019. The patients were grouped according to lymph node metastases and tumor-free and overall survival rates which were investigated with the Kaplan-Meier analysis. The logistic regression models were used for multivariate analysis to determine the risk factors for lymph node metastases. And the X-tile program was used to calculate the cutoff values for the lymph node parameters that discriminated survival.

**Results:**

The 1-year, 3-year, and 5-year overall survival rates of patients with distal cholangiocarcinoma after pancreatoduodenectomy were 75.2%, 37.1%, and 31.5%, respectively. And the 1-year, 3-year, and 5-year overall survival rates of patients without and with lymph node metastasis were 83.0%, 50.7%, and 42.5% and 63.5%, 19.0%, and 19.0% (*p* = 0.000), respectively. Logistic regression showed CA19-9 and portal vein system invasion as independent risk factors for lymph node metastases. The receiver operating characteristic curve showed the optimal cutoff value of CA19-9 to predict the lymph node metastases was 75.5 U/mL. Determined by the X-tile software, the optimal cutoff values of the lymph node dissection number were 24 (*p* = 0.021), the lymph node metastasis number were 1 and 7 (*p* = 0.504), and the lymph node metastasis rate were 0.13 (*p* = 0.002).

**Conclusion:**

Lymph node metastasis is an important factor affecting the long-term survival of distal cholangiocarcinoma patients.CA19-9 and portal vein system invasion are independent risk factors for lymph node metastasis. Besides, the lymph node dissection number and lymph node metastasis rate can predict the long-term survival better than lymph node metastasis number.

## Introduction

Cholangiocarcinoma is a rare malignancy and accounts for 2% of all malignancies [1]. Distal cholangiocarcinoma refers to the extrahepatic cholangiocarcinoma in which the tumor is below the confluence of the cystic duct, accounting for 20–40% of all cholangiocarcinoma [2, 3]. Surgical resection by pancreaticoduodenectomy remains the only definitively curative therapy for the long-term survival of distal cholangiocarcinoma patients [4]. And the 5-year survival rate of distal cholangiocarcinoma after pancreatoduodenectomy ranged from 22 to 47% [5–7]. Lymph node metastasis is widely accepted as an important survival factor for distal cholangiocarcinoma patients [8, 9]. Therefore, an optimal lymph node classification system is very important to predict the long-term prognosis of patients. In the latest American Joint Committee on Cancer (AJCC) TNM classification system for distal cholangiocarcinoma, the number of lymph node metastases has been adopted for classification, with N0 indicates zero, N1 indicates one to three, and N2 indicates four or more regional lymph node metastases which also suggests the dissection number of lymph node should not be less than 12. But some studies have suggested that the ratio of lymph node metastasis can refine staging and associate with the long-term survival of distal cholangiocarcinoma patients. However, due to the lack of studies, the value of these lymph node parameters is still controversial.

Therefore, we collectively reviewed 123 patients with distal cholangiocarcinoma after pancreaticoduodenectomy in our hospital to investigate the prognostic impact of lymph node metastasis and confirm the correlation between lymph node parameters (lymph node dissection number, lymph node metastasis number, and lymph node metastasis rate) and long-term prognosis.

## Patients and methods

### Patients

A retrospective analysis was made of 123 consecutive patients with distal cholangiocarcinoma who underwent pancreatoduodenectomy from January 2011 to December 2019 in the Department of Hepatobiliary Surgery of our hospital. All of the patients were diagnosed with adenocarcinoma according to the pathology, with no invasion of major celiac arteries (hepatic artery/celiac trunk/superior mesenteric artery) or distant metastasis.

The patients included 72 (58.5%) males and 51 (41.5%) females, and the age of the patients ranged from 29 to 84 (mean, 64.9 ± 9.2 years old). Forty-two (34.1%) patients underwent percutaneous transhepatic cholangial drainage (PTCD) before the operation, and 16 (13.0%) patients underwent endoscopic retrograde cholangiopancreatography (ERCP) before the operation. The radiological information was acquired from the latest CT or MRI examination before the operation.

This study has already been approved by the Ethics Committee of Beijing Chaoyang Hospital (acceptance number 2020-D-139). All procedures in this study involving human participants were performed in accordance with the ethical standards of the institutional research committee and the 1964 Helsinki Declaration.

### Treatment and follow-up

All of the patients underwent pancreatoduodenectomy. And regional lymph node dissection was performed routinely during the operation, including nodes along the common hepatic artery, nodes in the hepatoduodenal ligament, and anterior or posterior pancreatoduodenal nodes. In our center, the diagnosis of lymph node metastasis is completed by the pathologists. HE staining is the most commonly used method in the diagnosis of lymph node metastasis. If HE staining cannot make a definite diagnosis, further immunohistochemical staining should be carried out to confirm, mainly including CK7, CK19, and CEA. And if the immunohistochemical result is positive, lymph node metastasis will be diagnosed. To ensure the diagnostic accuracy, two chief physicians in the Department of Pathology provide independent diagnostic opinions, and a third chief physician participates in the diagnosis if the first two physicians disagree.

Patients were followed up by outpatient and telephone. They accepted blood routine and blood biochemistry examination every 1 month, carbohydrate antigen 19-9 (CA19-9) and CT scan every 3 months for 2 years, then CA19-9 and CT scan every 6 months above 2 years.

### Statistical analysis

Cataloged data included general conditions (gender, age, diabetes history, smoking history, biliary drainage history, leukocyte, neutrophil ratio, alanine aminotransferase (ALT), aspartate aminotransferase (AST), total bilirubin (TB), direct bilirubin (DB), glutamyltranspeptidase (GGT), albumin (ALB), CA19-9), pathological conditions (maximum tumor diameter, tumor differentiation degree, perineural invasion, portal vein system invasion, T stage, lymph node parameters), tumor recurrence, and patient survival.

The endpoint for the current analysis was patient death or tumor recurrence. The normal distribution data was represented by mean ± SD, and comparisons were analyzed with the Student *t* test according to data distribution. The non-normal distribution data was represented by M (P25, P75), and comparisons were analyzed with the Mann-Whitney rank-sum test. Categorical variables were presented as number and percentage and analyzed by the chi-square test or Fisher exact test, as appropriate.

Overall and tumor-free survival rates were calculated using the Kaplan-Meier method, and the log-rank test was used to compare the survival rate. Logistic regression models were used for multivariate analysis. Those variables, which were found to be significant in univariate analysis, were further enrolled in the multivariate analysis.

Using X-tile 3.6.1 software [10] to determine the best critical value of variables, *p* < 0.05 was considered to be statistically significant. The statistical measurements were performed using the SPSS 19.0 software program.

## Results

### Perioperative outcomes

All of the patients were successfully operated. The amount of bleeding was 500 (400, 600) mL, 43 patients received blood transfusion, and the operation time ranged from 6 to 15 h (mean, 9.8 ± 1.9 h). Moreover, the R0 resection is in 117 patients with an R0 rate of 95.1%, and the tumor size ranged from 0.5 to 6.5 cm (mean, 2.2 ± 1.0 cm). The lymph node dissection number ranged from 3 to 45 (mean, 18.8 ± 8.7). And the lymph node metastases are in 51 patients with a rate of 41.5%. The lymph node metastasis number of lymph node metastasis patients ranged from 1 to 11 (mean, 3.2 ± 2.6). In our group, 6 patients died during the perioperative period, and the perioperative mortality was 4.9%. The causes of deaths included 3 hemorrhage cases, 1 myocardial infarction case, 1 pulmonary embolism case, and 1 cerebral hemorrhage case. There were 34 patients with postoperative complications, and the incidence rate was 27.6%. Among them, biochemical fistulas happened in 14 (11.4%) cases, grade B pancreatic fistulas happened in 7 (5.7%) cases, grade C pancreatic fistulas happened in 5 (4.1%) cases, and hemorrhages happened in 9 (7.3%) cases.

### Prognostic outcomes

**Fig. 1:**
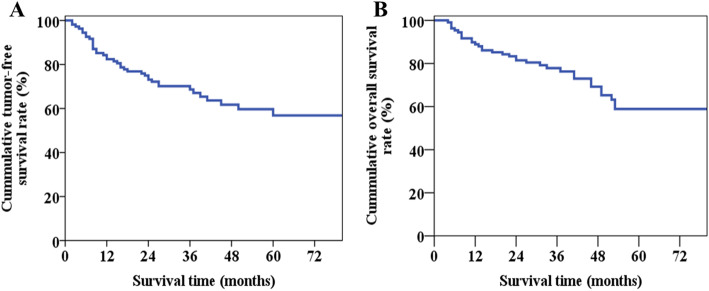
The tumor-free (**a**) and overall (**b**) survival curves for all patients

The final follow-up date is April 2020, and the median follow-up length is 41.0 months. Two patients missed the follow-up. Finally, 115 patients were included in the study. The 1-year, 3-year, and 5-year tumor-free and overall survival rates of all patients were 67.0%, 36.0%, and 27.1% and 75.2%, 37.1%, and 31.5%, respectively (Fig. 1a, b). The median tumor-free and overall survival times were 38.2 and 44.7 months, respectively.

**Fig. 2:**
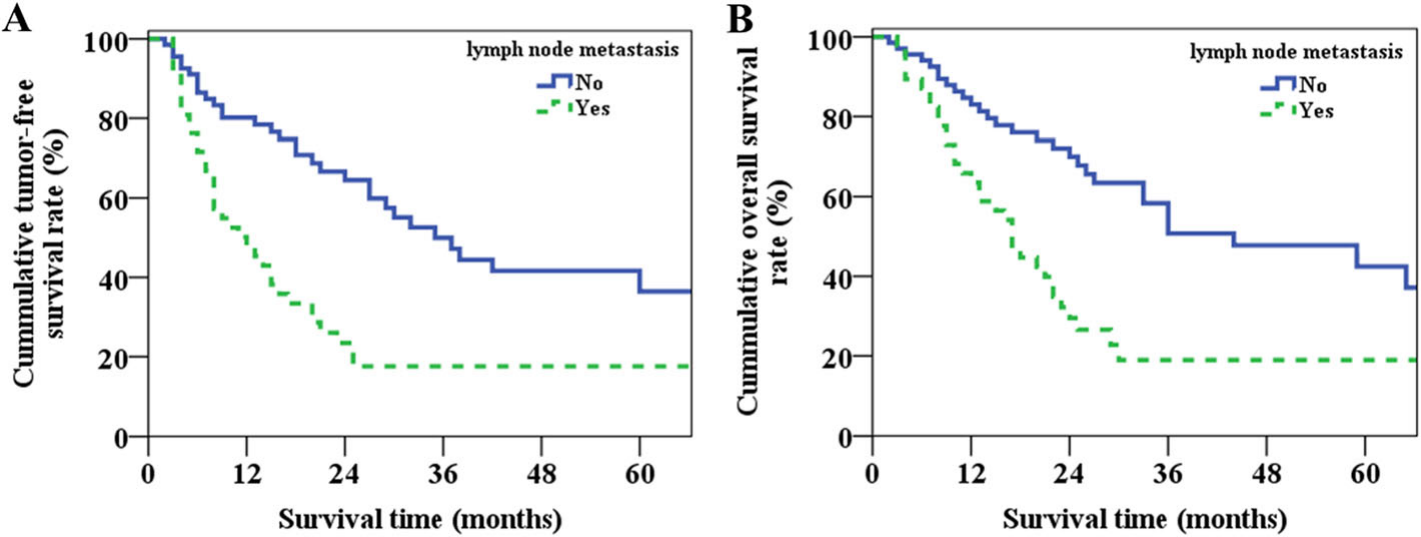
The tumor-free (**a**) and overall (**b**) survival curves for patients without and with lymph node metastasis

The 1-year, 3-year, and 5-year tumor-free survival rates of patients without and with lymph node metastasis were 80.1%, 50.0%, and 36.4% and 47.7%, 17.6%, and 17.6%, respectively (*p* = 0.000, Fig. 2a). And the 1-year, 3-year, and 5-year overall survival rates were 83.0%, 50.7%, and 42.5% and 63.5%, 19.0%, and 19.0% (*p* = 0.000, Fig. 2b). The median tumor-free and overall survival time of patients without and with lymph node metastasis were 48.3, 55.5, and 23.4, 27.5 months, respectively.

### Risk factors of lymph node metastasis

In the 123 patients, 51 patients were found lymph node metastases. Univariate analysis identified ALB, DB, CA19-9, differentiation, and portal vein system invasion as the risk factors for lymph node metastases (Table 1). And logistic regression showed CA19-9 (RR = 7.064, 95% CI 2.489~20.051) and portal vein system invasion (RR = 4.610, 95% CI 1.252~16.972) as independent risk factors for lymph node metastases (Table 2).

**Table 1:** Risk factors for lymph node metastases by univariate analysis

Variables	Lymph node metastasis group (*n* = 51)	Control group (*n* = 72)	*p* value
Age (years)	65.1 ± 10.6	64.9 ± 8.1	0.888
Leukocyte (× 10^9^)	6.8 ± 2.6	6.8 ± 2.0	0.880
Neutrophil ratio (%)	67.6 ± 10.7	66.0 ± 10.1	0.408
ALB (g/L)	32.8 ± 5.8	35.0 ± 6.1	0.047
ALT (U/L)	73.0 (35.0, 157.7)	67.0 (33.8, 146.3)	0.904
AST (U/L)	67.0 (34.5, 121.0)	58.0 (39.3, 92.5)	0.698
TB (μmol/L)	165.6 (43.0, 246.7)	76.6 (32.2, 195.6)	0.102
DB (μmol/L)	128.8 (29.6, 205.5)	70.8 (23.7, 133.7)	0.028
GGT (U/L)	364.0 (166.0, 726.0)	386.0 (127.0, 1005.0)	0.679
CA19-9 (U/mL)	176.6 (56.5, 407.3)	36.9 (12.7, 71.6)	0.000
Tumor diameter (cm)	2.3 ± 1.1	2.1 ± 0.9	0.350
Gender (%)			0.491
Male	28 (54.9)	44 (61.1)	
Female	23 (45.1)	28 (38.9)	
Diabetes history (%)			0.436
Yes	16 (31.4)	18 (25.0)	
No	35 (68.6)	54 (75.0)	
Smoking history (%)			0.374
Yes	18 (35.3)	20 (27.8)	
No	33 (64.7)	52 (72.2)	
Biliary drainage history (%)			0.986
Yes	24 (47.1)	34 (47.2)	
No	27 (52.9)	38 (52.8)	
Differentiation (%)			0.017
Poor	9 (17.6)	20 (27.8)	
Moderate	20 (39.2)	38 (52.8)	
Well	22 (43.1)	14 (19.4)	
Perineural invasion (%)			0.215
Yes	47 (92.2)	61 (84.7)	
No	4 (7.8)	11 (15.3)	
Portal vein system invasion (%)			0.002
Yes	13 (25.5)	4 (5.6)	
No	38 (74.5)	68 (94.4)	
T stage (%)			0.946
T1	1 (2.0)	1 (1.3)	
T2	5 (9.8)	8 (11.1)	
T3	45 (88.2)	63 (87.5)

**Table 2:** Risk factors for lymph node metastases by logistic regression

Variables	RR	95% CI	*p* value
ALB	0.469	0.191~1.148	0.097
DB	0.574	0.171~1.923	0.368
CA19-9	7.064	2.489~20.051	0.000
Differentiation	1.727	0.942~3.165	0.077
Portal vein system invasion	4.610	1.252~16.972	0.013

**Fig. 3:**
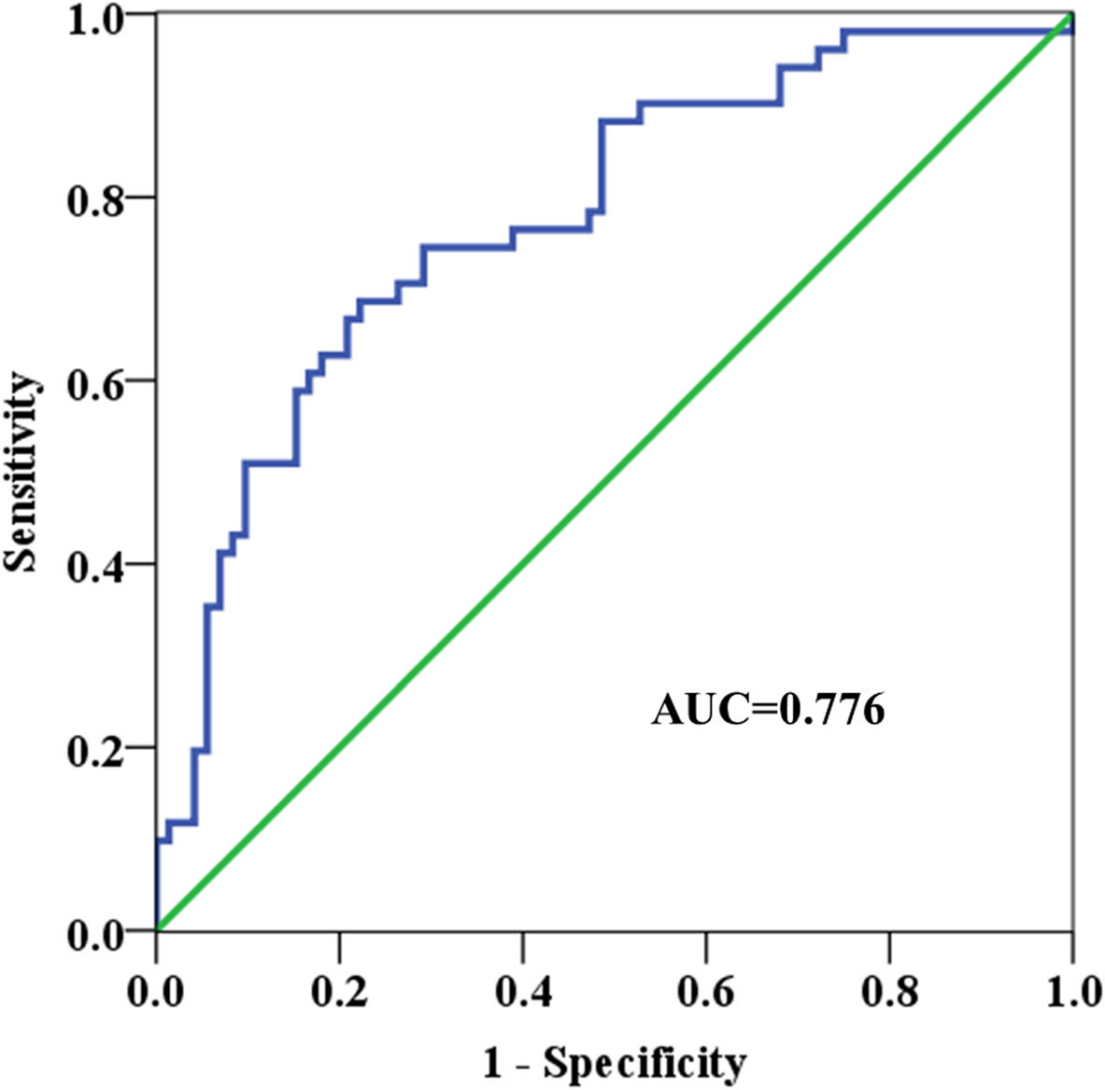
The receiver operating characteristic curve of CA19-9 to predict the lymph node metastases

In logistic regression analysis, we found that there were significant differences in variables, and ROC curve analysis was used to further analyze to determine the optimal critical value. The optimal cutoff value of the CA19-9 was 75.5 U/mL to predict the lymph node metastases (area under the curve (AUC) was 0.776, and 95% CI was 0.692–0.861, Fig. 3). The sensitivity and specificity to diagnose lymph node metastasis were 68.6% and 77.8%, respectively.

### Prognostic of lymph node parameters

**Fig. 4:**
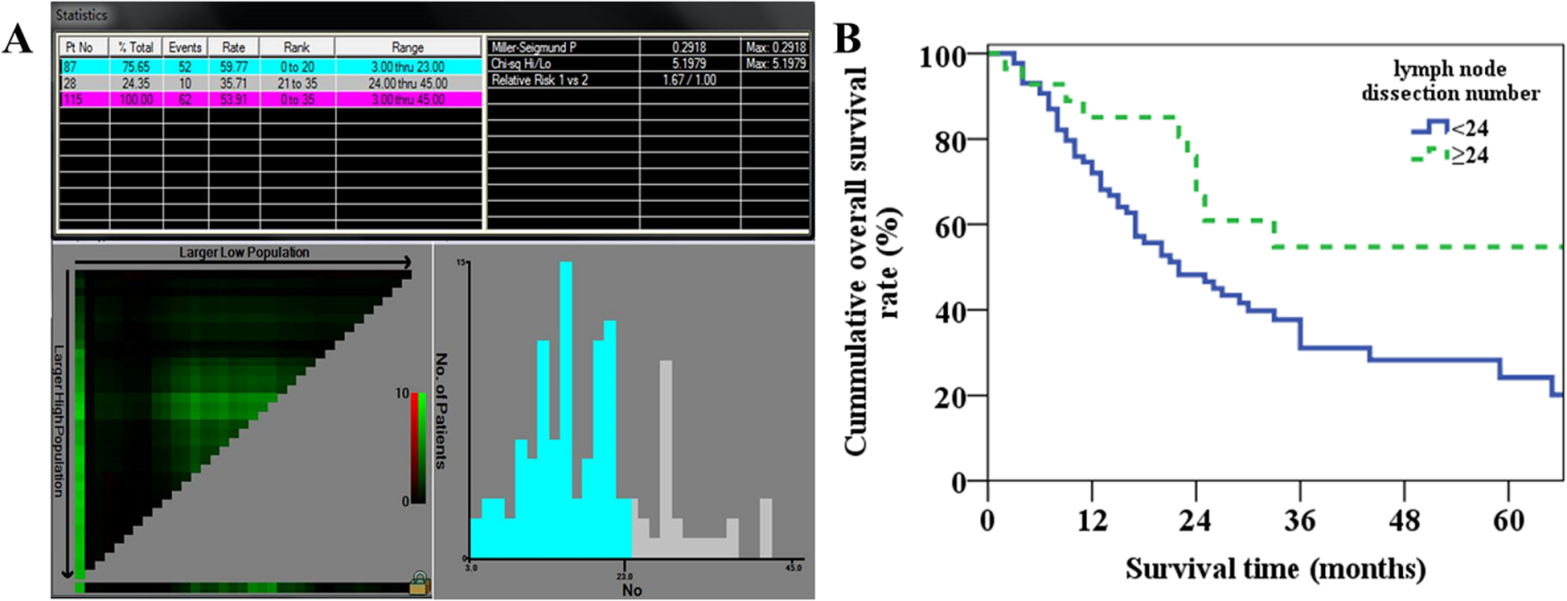
The X-tile software determined the optimal cutoff value of the lymph node dissection number was 24 (**a**) and overall survival curves (**b**) for patients with lymph node dissection number of ≥ 24 and < 24

The lymph node dissection number ranged from 3 to 45 (mean, 18.8 ± 8.7) for the 115 patients with integral follow-up data. The X-tile software determined the optimal cutoff value of the lymph node dissection number was 24 (Fig. 4a). The 1-year, 3-year, and 5-year overall survival rates of patients with lymph node dissection number ≥ 24 and < 24 were 85.1%, 54.8%, and 54.8% and 72.0%, 31.0%, and 24.2% (*p* = 0.021, Fig. 4b), respectively.

**Fig. 5:**
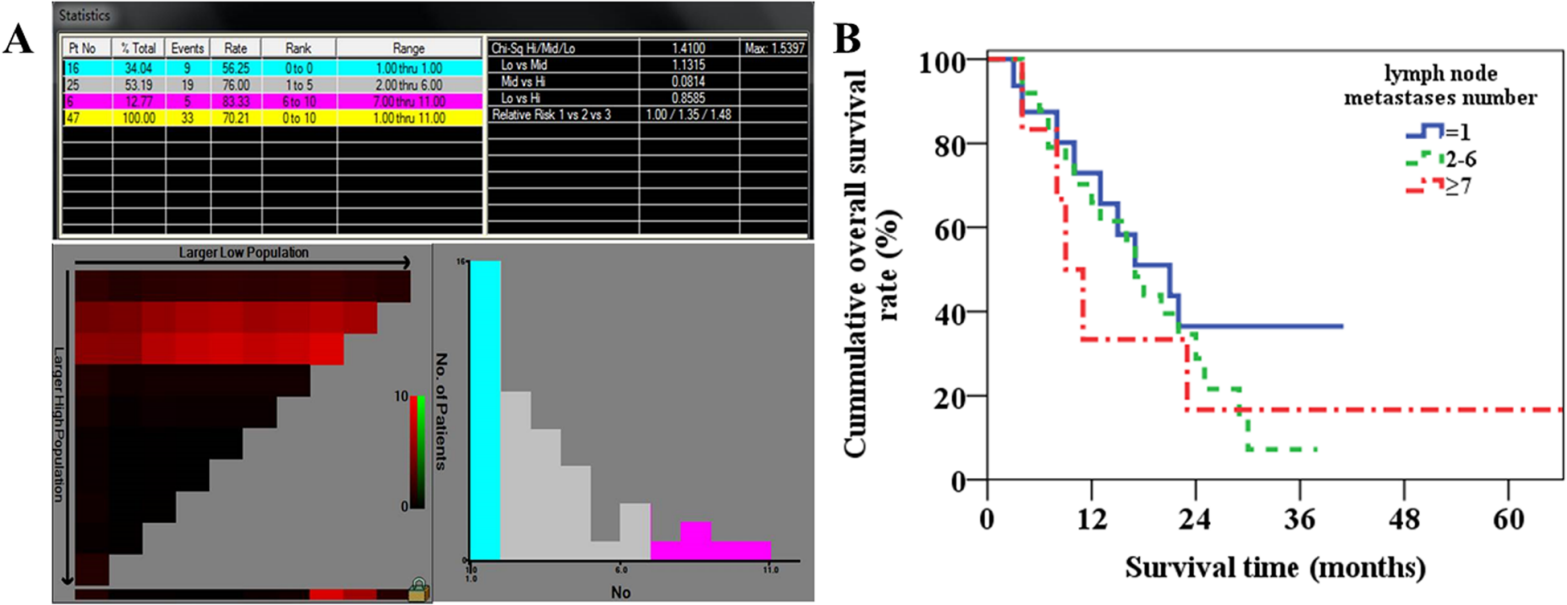
The X-tile software determined the optimal cutoff values of the lymph node metastasis number were 1 and 7 (**a**) and overall survival curves (**b**) for patients with lymph node dissection number of 1, 2 to 6, and ≥ 7

The lymph node metastasis number ranged from 1 to 11 (mean, 3.2 ± 2.6) for the 47 lymph node metastasis patients with integral follow-up data. The X-tile software determined the optimal cutoff values of the lymph node metastasis number were 1 and 7 to the N1 patients (Fig. 5a). The 1-year and 3-year overall survival rates of patients with lymph node metastasis number = 1, 2 to 6, ≥ 7 were 72.9%, 65.9%, and 33.3% and 36.5%, 7.2%, and 16.7% (*p* = 0.504, Fig. 5b), respectively.

**Fig. 6:**
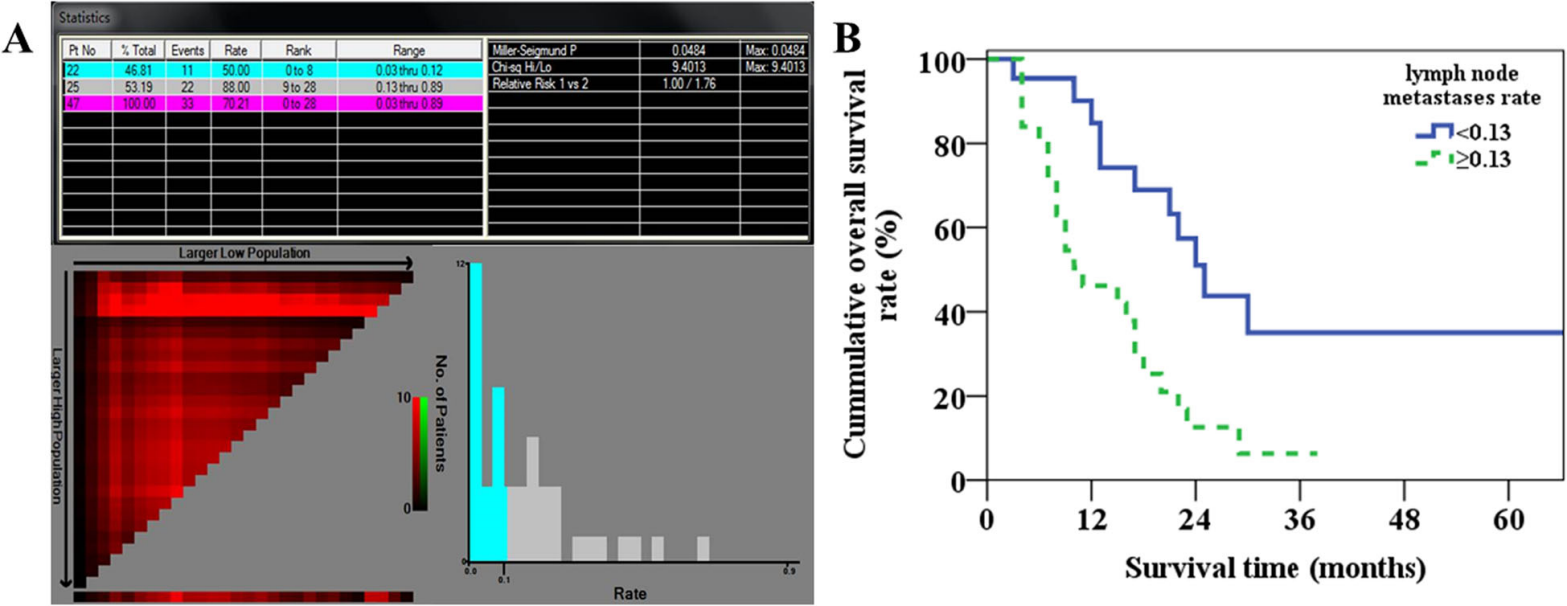
The X-tile software determined the optimal cutoff value of the lymph node metastasis rate was 0.13 (**a**) and overall survival curves (**b**) for patients with lymph node metastasis rate of < 0.13 and ≥ 0.13

The lymph node metastasis rate ranged from 0.03 to 0.89 (mean, 0.19 ± 0.17) of the 47 lymph node metastasis patients with integral follow-up data. The X-tile software determined the optimal cutoff value of the lymph node metastasis rate was 0.13 to the N1 patients (Fig. 6a). The 1-year and 3-year overall survival rates of patients with lymph node metastasis rate < 0.13 and ≥ 0.13 were 84.8% and 46.2%, and 35.0% and 6.3% (*p* = 0.002, Fig. 6b), respectively.

## Discussion

At present, the optimum treatment of distal cholangiocarcinoma is still based on surgery, and pancreatoduodenectomy is the standard operation [11, 12]. Andrianello et al. [13] reviewed the data of 1490 cases of distal cholangiocarcinoma after pancreatoduodenectomy in the USA. The overall median survival time was 31 months, and the 1-year and 3-year survival rates were 89% and 40%, respectively. Our research showed the 1-year and 3-year survival rates were 75.2% and 37.1% respectively.

For surgical patients, it is known to all that lymph node metastasis is one of the most important risk factors for long-term survival [14, 15]. Kiriyama et al. [16] reviewed the data of 370 patients with distal cholangiocarcinoma that underwent pancreatoduodenectomy in 24 hospitals in Japan. The 3-year survival rate of patients without lymph node metastasis was significantly higher than that of patients with lymph node metastasis (66.3% vs 36.1%, *p* < 0.001). Byrling et al. [17] showed that lymph node metastasis was the only independent risk factor for long-term survival of patients with distal cholangiocarcinoma (*R* = 2.88, *p* = 0.016). Our research also showed that the long-term prognosis of patients without lymph node metastasis was better than that of patients with lymph node metastasis (*p* = 0.000). Therefore, accurate preoperative evaluation of lymph node metastasis is very important for predicting the long-term prognosis.

At present, the preoperative diagnosis and evaluation of distal cholangiocarcinoma mainly relied on imaging examination; it was commonly used for judging whether there is lymphadenopathy or not, which value was limited in the diagnosis of lymph node metastasis. CA19-9 is a serological marker commonly used in clinical diagnosis and prognosis of cholangiocarcinoma [18]. Lumachi et al. [19] showed that the sensitivity and specificity of CA19-9 for the diagnosis of cholangiocarcinoma were 74% and 82%, respectively. Tella et al. [20] reviewed the data of 2100 patients with extrahepatic cholangiocarcinoma in the National Cancer Database of the USA, and CA19-9 was increased in 1474 (70.2%) patients. The median survival time of patients with increased CA19-9 was significantly lower than that of normal patients (8.5 months vs 16.0 months, *p* < 0.01), and it also showed CA19-9 was an independent risk factor for the long-term survival (RR = 1.72, 95% CI 1.46–2.02). Moreover, our data showed that CA19-9 was also significantly correlated with lymph node metastasis, which may be the cause of poor long-term survival in patients with increased CA19-9, especially in the patients with CA19-9 higher than 75.5 U/mL.

On the other hand, our data showed that portal vein system invasion was another independent risk factor for lymph node metastasis. We think it may be related to the behavior of the cholangiocarcinoma, because the distal common bile duct is adjacent to the lymphatics and portal vein system. Therefore, patients with distal cholangiocarcinoma are also prone to lymph node metastasis in the event of portal vein system invasion. Miura et al. [21] reviewed the data of 129 patients with distal cholangiocarcinoma who underwent pancreatoduodenectomy, and the study showed that the lymph node metastasis rate of patients with portal vein invasion was significantly higher than that of patients without vascular invasion (87% vs 37%, *p* = 0.005). Maeta et al. [22] reviewed the data of 453 patients with distal cholangiocarcinoma in 31 centers. The lymph node metastasis rate of patients with portal vein invasion was 94%, significantly higher than that of patients without portal vein invasion (60.7%, *p* < 0.001).

Therefore, pancreatoduodenectomy with thorough lymph node dissection is the key to improve the long-term survival of patients with distal cholangiocarcinoma. The 8th AJCC TNM classification system for distal cholangiocarcinoma has suggested the dissection number of lymph node should not be less than 12 and has divided the lymph node metastasis patients into N1 and N2 with the cutoff value of 4. Ito et al. [23] reviewed the data of 113 patients with distal cholangiocarcinoma who underwent pancreatoduodenectomy and found that the number of lymph nodes should not be less than 12 (*p* = 0.008). Murakami et al. [24] showed that lymph node metastasis was an independent risk factor for the long-term survival of patients with distal cholangiocarcinoma (*p* = 0.043), and further analysis showed the optimal cutoff value for lymph node metastasis number was 2 (*p* < 0.001). But our study showed a different outcome that the optimal cutoff value of the lymph node dissection number was 24 and the optimal cutoff values of the lymph node metastasis number were 1 and 7 to the N1 patients. Besides, our study still showed the lymph node metastasis number was not related to the long-term survival.

There were still other researches which showed that the number of lymph node dissection and lymph node metastases were not related to the long-term survival, but lymph node metastasis rate was an independent risk factor. A retrospective study by Oshiro et al. [25] showed that the lymph node dissection number being 12 as the cutoff value did not affect the long-term survival of patients with distal cholangiocarcinoma (*p* = 0.484), while the lymph node metastasis rate being 0.2 as the cutoff value was an independent risk factor for the prognosis (*p* = 0.023). Kiriyama et al. [16] also showed that the median survival time of patients with lymph node metastasis rate < 0.17 was significantly better than those with lymph node metastasis rate ≥ 0.17 (2.3 years vs 1.4 years, *p* = 0.002). Our study showed that the optimal cutoff value of lymph node metastasis rate was 0.13 of the N1 patients, which meant that surgeons had to examine at least 8 lymph nodes in order to accurately estimate the prognosis. Lymph node metastasis is the commonest form of distal cholangiocarcinoma metastasis, and regional lymph node dissection is the standard surgical procedure for distal cholangiocarcinoma; therefore, a sufficient number of lymph node dissection must be ensured to prevent missed diagnosis. However, distal cholangiocarcinoma may cause biliary obstruction, jaundice, cholangitis, and other symptoms. Regional lymph node hyperplasia may occur, but most of these lymph nodes are caused by inflammatory response without tumor metastasis. If a large number of lymph nodes are dissected, it will be biased to judge the prognosis simply by the lymph node dissection number or metastasis number. The lymph node metastasis rate can reflect whether there is lymph node metastasis and also can reflect the severity of lymph node metastasis by proportion. Therefore, for patients with lymph node metastasis, it is more likely to reflect the aggressiveness of the tumor compared with the lymph node dissection number or metastasis number and may have more clinical values in the prediction of the prognosis.

The limitation of this study is that the effect of hyperbilirubinemia on CA19-9 cannot be excluded, so the optimal cutoff value of CA19-9 for lymph node metastasis needs to be further verified by large sample data. And our study is retrospective, so the operating surgeon is not the only one.

The limitation of our study is due to the single-center retrospective design and the sample of patients that is not large. And the operating surgeon is not within 9 years. These limitations may affect the results, which need to be further confirmed by large and multicenter researches.

In conclusion, lymph node metastasis is an important factor affecting the long-term survival of patients with distal cholangiocarcinoma, and CA19-9 and portal vein system invasion are independent risk factors for lymph node metastasis. The lymph node dissection number and lymph node metastasis rate can predict the long-term survival better than lymph node metastasis number.

## Supplementary information

**Additional file 1:.** Ethics approval.

## Data Availability

We do not wish to share our data because it is personal. We can provide it if the editor needs it, but we refuse to share the data publicly.
