# Supplementary material for: Prognostic impact of lymph node parameters in distal cholangiocarcinoma after pancreaticoduodenectomy
Source: World J Surg Oncol. 2020 Oct 8;18:262. doi: 10.1186/s12957-020-02040-1 (PMC7545845; doi:10.1186/s12957-020-02040-1)
Supplement: Supplementary file 1 — Additional file 1:. Ethics approval. [file 12957_2020_2040_MOESM1_ESM.pdf]

首都医科大学附属北京朝阳医院动物福利与伦理工作委员会
科研课题申报快速审查批件（1.0版）

受理号： 2020-3-17-139　　　　编号： 2020-科-139

科研项目名称	Prognostic impact of lymph node parameters in distal cholangiocarcinoma after pancreaticoduodenectomy		
申报课题类型	其它		
我院项目负责人	吕少诚		
项目负责人所在科室	肝胆外科		
报送材料	科研项目伦理审查申请表		
	项目申请书		
	知情同意书		
主审委员	顾佳		
主审结果	同意（1）票	不同意（0）票	作必要修改后同意（0）票
审查决定	■同意 □作必要修改后同意 □不同意		

审查意见：
无

注意事项：
1. 本申报审查批件将在伦理办公室备案。
2. 请在项目获得有关部门批准并在我院正式立项后重新递交伦理审查资料，经我院伦理委员会会议审查批准后方可开展研究。

主任委员/副主任委员签字：

动物福利与伦理工作委员会（盖章）

首都医科大学附属北京朝阳医院 医学伦理委员会

2020年4月7日

地址：北京市朝阳区工人体育场南路8号　邮政编码：100020　电话：010-85231720或85231204
